# Don’t

**Published:** 1879-07

**Authors:** 


### DON'T.

The family whose income exceeds fifteen hundred dollars a year is too apt to assume the airs of those who have as many thousands. As hot weather approaches, they sigh for a vacation at some fashionable watering place or at the seaside. The baby suddenly grows thin and fretful, the mother nervous and "run down," while father, even, thinks a vacation would do him good. Singularly enough, their thoughts all turn toward some fashionable resort,—some springs, mountain-house or sea-beach. It never enters their heads that a vacation can be had near home, at one quarter the expense, but where fashion leads there they must go. Don't!

Procure a tent, go to some convenient woods, by a brook and near a farm house, and there "camp out," with wife and children, employing your time in reading, fishing, painting and gathering wild flowers, and capturing butterflies and other insects. Why, any woods will afford you endless amusement if you will but look for it. You will be astonished to see how many varieties of flowers and grasses are growing about that you never had any knowledge of before. The birds will delight you with their plumage and melodies, and the beautiful creeping creatures in the way of moths and "bugs" will interest you by the hour with their habits and gaudy colors.

Sit down in the woods anywhere and study Nature. She is ever ready to entertain you. We have had more genuine pleasure in watching a sparrow feed her young,—a colony of ants building a home,—a kingfisher diving for minnows, or a butterfly bursting his chrysalid shell, than was ever had from a sojourn of a week at any fashionable summering resort.

